# Invasive laser acupuncture vs. electroacupuncture for non-specific chronic low back pain: protocol for a randomized clinical trial

**DOI:** 10.3389/fmed.2025.1659696

**Published:** 2025-10-30

**Authors:** Yejin Hong, Dongwoo Nam, Changsop Yang, Byoung-Kab Kang, Ae-Ran Kim, Kyung Min Shin, Jihye Kim, Saerom Jeon, Gwang-Cheon Park, Jeong-Cheol Shin, Jae-Hong Kim

**Affiliations:** ^1^Department of Acupuncture and Moxibustion, Kyung Hee University Korean Medicine Hospital, Seoul, Republic of Korea; ^2^Department of Acupuncture and Moxibustion, College of Korean Medicine, Kyung Hee University, Seoul, Republic of Korea; ^3^KM Science Research Division, Korea Institute of Oriental Medicine, Daejeon, Republic of Korea; ^4^R&D Strategy Division, Korea Institute of Oriental Medicine, Daejeon, Republic of Korea; ^5^Digital Health Research Division, Korea Institute of Oriental Medicine, Daejeon, Republic of Korea; ^6^Korean Medicine Research, Seoul, Republic of Korea; ^7^Clinical Research Center, Dongshin University Gwangju Korean Medicine Hospital, Gwangju, Republic of Korea; ^8^Department of Acupuncture and Moxibustion Medicine, College of Korean Medicine, Dongshin University, Naju, Republic of Korea

**Keywords:** chronic low back pain, invasive laser acupuncture, low level laser therapy, electroacupuncture, randomized clinical trial

## Abstract

**Background:**

This study aims to evaluate the short-term efficacy of 650 nm invasive laser acupuncture (ILA) compared to conventional electroacupuncture (EA) in reducing pain in patients with non-specific chronic low back pain (NSCLBP).

**Method:**

This is a prospective, multicenter, randomized, single-blind, controlled trial. Ninety patients with NSCLBP will be recruited and randomly assigned (1:1) to receive either 650 nm ILA or EA. Treatments will be administered at the bilateral acupoints BL23, BL24, BL25, and GB30 for 10 min per session, twice weekly for 4 weeks (8 sessions in total). The primary outcome is the change in the Visual Analog Scale (VAS) score 1 week after treatment completion. Secondary outcomes include VAS scores at interim and follow-up time points, Oswestry Disability Index, European Quality of Life Five Dimension Five-Level Scale, and the proportion of responders. Safety assessments and adverse event monitoring will be conducted throughout the trial.

**Conclusion:**

This multicenter randomized controlled trial compares the effects of ILA and EA with the change in VAS as a primary efficacy endpoint in 90 patients with NSCLBP. This findings will provide clinical evidence of the comparative efficacy and safety of 650 nm ILA versus EA in patients with NSCLBP, supporting its potential as a viable non-pharmacological treatment option.

**Clinical trial registration:**

https://cris.nih.go.kr/cris/search/detailSearch.do?search_lang=E&focus=reset_12&search_page=M&pageSize=10&page=undefined&seq=29960&status=5&seq_group=29960, identifier KCT0010475.

## 1 Introduction

Low back pain (LBP) is pain or discomfort that occurs between the subcostal line and the buttock crease (1), it is considered a symptom like headache or dizziness, rather than an independent disease (2). LBP is commonly classified as a specific spinal disorder, radiculopathy, or non-specific LBP, depending on the underlying cause (1, 3, 4). Approximately 90% of cases are regarded as non-specific low back pain, and no definitive etiological factors can be identified (5). In addition, LBP is categorized as acute (within 4 weeks), subacute (4–12 weeks), and chronic (more than 12 weeks) based on symptom duration (6), and non-specific chronic low back pain (NSCLBP) is defined as pain that persists without severe lesions (1–4, 7). Chronic pain is considered an important clinical problem because it leads to a reduced quality of life and substantial socioeconomic burden.

According to both domestic and international clinical practice guidelines, including the 2023 World Health Organization (WHO) guideline for NSCLBP, non-pharmacological interventions are strongly recommended as first-line treatment options alongside conventional pharmacological approaches such as non-steroidal anti-inflammatory drugs. These recommended strategies include acupuncture, spinal manipulative therapy, and structured exercise programs have demonstrated efficacy comparable to pharmacological interventions but with fewer adverse effects. In addition, various other non-pharmacological approaches, including low-level laser therapy (LLLT), multidisciplinary rehabilitation, and psychosocial interventions are also recommended as part of a comprehensive management strategy (2–4, 6–10).

LLLT is attracting attention as an alternative treatment and has been the focus of ongoing research because it relieves pain by promoting cell metabolism, improving blood flow, and inducing anti-inflammatory effects (11–13). Among the laser wavelengths commonly used in pain management (650–900 nm), the 650 nm wavelength has demonstrated optimal penetration and therapeutic effects in musculoskeletal disorders (13). However, as laser light penetrates the body, its energy is attenuated because of reflection, scattering, and absorption, limiting its ability to reach deep tissues and posing a challenge in stimulating acupoints situated beneath the skin and subcutaneous fat (12, 14). To address these limitations, alternative approaches such as invasive laser acupuncture (ILA) are being explored to enable more effective delivery of laser energy to deeper tissue layers.

ILA was developed as a novel technique to overcome the limitations of conventional laser acupuncture (LA) by allowing direct delivery of laser energy to deeper acupoints (15–18). This method utilizes a sterile needle (diameter 0.3 mm, length 30 mm, inner diameter 0.15 mm) embedded with an optical fiber, which emits a 650 nm wavelength laser directly from the needle tip after insertion into the acupoint. By transmitting laser energy invasively, the ILA can penetrate energy to a deeper depth compared to conventional LLLT and minimize energy loss due to scattering or absorption from the skin barrier. In addition, it allows for the combined application of mechanical stimulation from acupuncture and the photobiomodulatory effects of LLLT, enabling effective and safe stimulation even at a low-power level.

In our previous exploratory randomized controlled trial (RCT) comparing 650 nm and 830 nm ILA with a sham laser, the 650 nm group showed significant improvements in pain intensity and functional disability (16). Subsequently, a confirmatory RCT using 650 nm ILA (650 nm wavelength, 50 Hz, 20 mW) demonstrated significant pain reduction in patients with NSCLBP compared with a sham laser (18). However, no studies have yet compared ILA with other active therapeutic interventions.

This clinical trial will evaluate the clinical efficacy of 650 nm ILA in patients with NSCLBP. In particular, we aim to explore the degree of superiority of ILA in pain relief compared with electroacupuncture (EA), which is widely used in clinical practice in Korean medicine. Thus, we provide a basis for the possibility of expanding non-pharmacological treatment strategies in the future.

## 2 Methods

### 2.1 Aim

This clinical trial aims to explore the superiority of the short-term pain relief effect of 650 nm wavelength ILA compared with that of EA stimulation in patients with NSCLBP.

### 2.2 Study design

This study is a multicenter, single-blind, RCT designed in accordance with the Standard Protocol Items: Recommendations for Interventional Trials (SPIRIT) and Consolidated Standards of Reporting Trials (CONSORT) 2010 guidelines. The trial will be conducted at the DongShin University Gwangju Korean Medicine Hospital and Kyung Hee University Korean Medicine Hospital in South Korea as a prospective, parallel-arm study.

**Figure 1:**
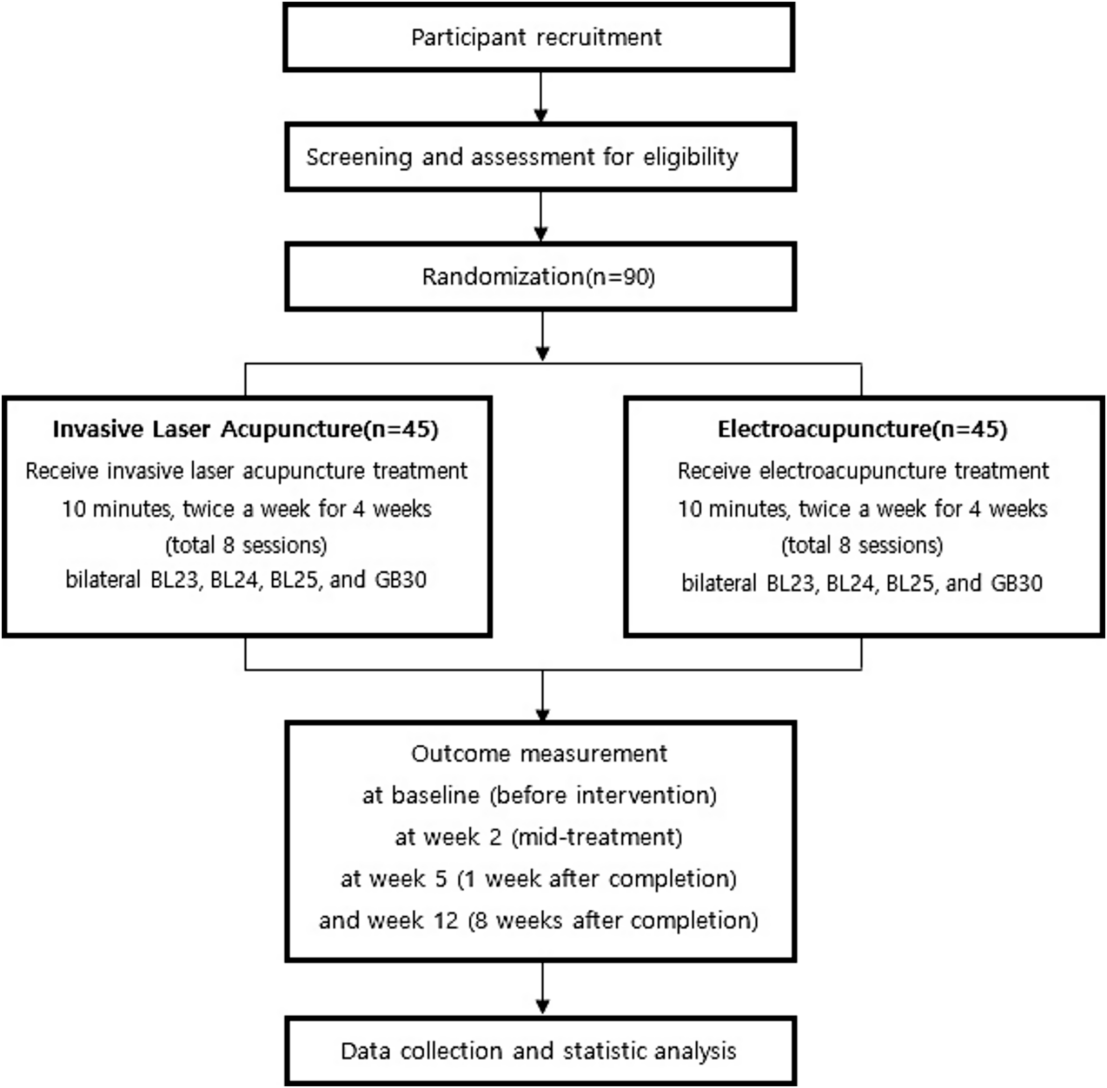
Study design flow chart. Flow diagram of the randomized controlled trial comparing invasive laser acupuncture (ILA) and electroacupuncture (EA) in 90 patients with non-specific chronic low back pain. Participants were randomly assigned to receive ILA or EA twice weekly for 4 weeks. Outcomes were assessed at baseline, mid-treatment (week 2), 1 week after completion (week 5), and 8 weeks after completion (week 12).

Ninety participants with NSCLBP will be randomly assigned in a 1:1 ratio to receive either 650 nm ILA or EA over a 4-week period. Treatments will be administered twice weekly for a total of eight sessions, targeting BL23, BL24, BL25, and GB30 bilaterally (Figure 1).

Clinical outcomes will be assessed at baseline (Visit 1), mid-treatment (Visit 5), 1 week post-treatment (Visit 9), and 8 weeks post-treatment (Visit 10). The primary outcome is the change in the Visual Analog Scale (VAS) score from baseline to 1 week after treatment completion. Secondary outcomes include changes in VAS, Oswestry Disability Index (ODI), and European Quality of Life Five Dimension Five Level scale (EQ-5D-5L) scores across all time points as well as the proportion of responders, defined as participants achieving at least a 30% reduction in VAS without increased analgesic use.

The clinical trial design is shown in Table 1.

**Table 1:** Schedule of enrollment, interventions, and assessments in accordance with the SPIRIT statement.

Procedures	Study period							
	Enrollment	Allocation	Post-allocation					Close-out
Timepoint	Screening		Visits 1–2	Visits 3–4	Visits 5–6	Visits 7–8	Visit 9	Visit 10
Time	Week		1	2	3	4	5 V8 + 1 week (±3 days)	12 V8 + 8 week (±3 days)
Enrollment								
Informed consent	●							
Sociodemographic profile	●							
Vital signs	●	●	●	●	●	●	●	●
Medical history	●							
Inclusion/exclusion criteria	●							
Allocation		●						
Visual analogue scale of pain	●							
Beck depression inventory-II test	●							
Interventions								
Invasive laser acupuncture or electroacupuncture			●	●	●	●		
Education on self-management and exercise			●	●	●	●		
Assessments								
Changes in medical history			●	●	●	●	●	●
Safety assessment (incidence of AEs)			●	●	●	●	●	●
Clinical laboratory tests	●						●	
Visual analogue scale of pain			●				●	●
Scores for the korean version of the oswestry disability index			●				●	●
European quality of life five-dimension five-level scale			●				●	●

### 2.3 Participants

#### 2.3.1 The inclusion criteria were as follows

Individuals aged 19–70 years; diagnosis of NSCLBP persisting for more than 3 months, with pain occurring at least 14 days per month during the preceding 3 months; moderate pain intensity at the time of screening, defined by a VAS score between 35 and 74 on a 100 mm scale (19); sufficient Korean language proficiency to complete the study assessments accurately; and provision of written informed consent indicating voluntary participation.

#### 2.3.2 The exclusion criteria were as follows

LBP with radiculopathy or neurological deficits; serious spinal conditions (e.g., malignancy, fracture, infection, cauda equina syndrome); major comorbidities (e.g., cancer, organ failure); psychiatric disorders or substance abuse within the past 6 months; other musculoskeletal or systemic diseases affecting the lower back (e.g., ankylosing spondylitis, fibromyalgia); contraindications to ILA (e.g., bleeding tendency, severe skin lesions, implanted metallic or electronic devices); moderate to severe depression (BDI-II ≥ 23) (20); lumbar surgery within the past year or scheduled during the study period; pregnancy or planned pregnancy; current participation in another trial; legal or compensation-related treatment; or any conditions deemed inappropriate by the investigator.

### 2.4 Study site

Recruitment, intervention, and follow-up for this study will be conducted at two research centers. Recruitment, treatment, and follow-up assessments will be conducted in the outpatient clinics of participating hospitals.

### 2.5 Methods of recruitment

To recruit participants for the clinical trial, advertisements will be placed in mass media, such as flyers and daily newspapers, as well as on bulletin boards and websites of the participating institutions. If recruitment is delayed, additional local advertisements will be implemented via subways, buses, apartment bulletin boards, clinical trial websites, or applications. Additionally, collaboration with online communities, local gatherings, and patient support groups for individuals with LBP will be sought to enhance outreach and engagement.

### 2.6 Randomization, allocation, and blinding

Participants will be assigned to either the 650 nm ILA group or the EA control group in a 1:1 ratio. Randomization will be performed by an independent statistician using SAS® version 9.4 (SAS Institute Inc., Cary, NC, United States) through stratified block randomization to ensure equal allocation probabilities across groups. A set of serial numbers corresponding to the randomization sequence will be generated in advance and uploaded to a secure system by a statistician not involved in the conduct or assessment of the trial. Serial numbers will be sealed in opaque envelopes and stored in double-locked cabinets. The investigator responsible for managing the serial numbers will open the envelopes sequentially and assign each participant to the practitioner who will administer the intervention. Given the inherent differences in procedural characteristics and sensory experiences between ILA and EA, blinding will be maintained for outcome assessors, co-investigators, data managers, and statisticians involved in data analysis.

### 2.7 Interventions and comparisons

Trained Korean medical doctors will perform the treatment, and investigators for the interventions will undergo joint training to ensure compliance with the study protocol. Treatments will be applied bilaterally at four acupuncture points: BL23, BL24, BL25, and GB30, which are commonly used for managing LBP (13–15). These acupoints are closely related to the lumbar paraspinal and gluteal muscles involved in spinal stabilization and pelvic motion, providing a biomechanical basis for their selection. Each session will last 10 min and will be administered twice weekly for 4 weeks, for a total of eight treatment sessions per participant.

**Figure 2:**
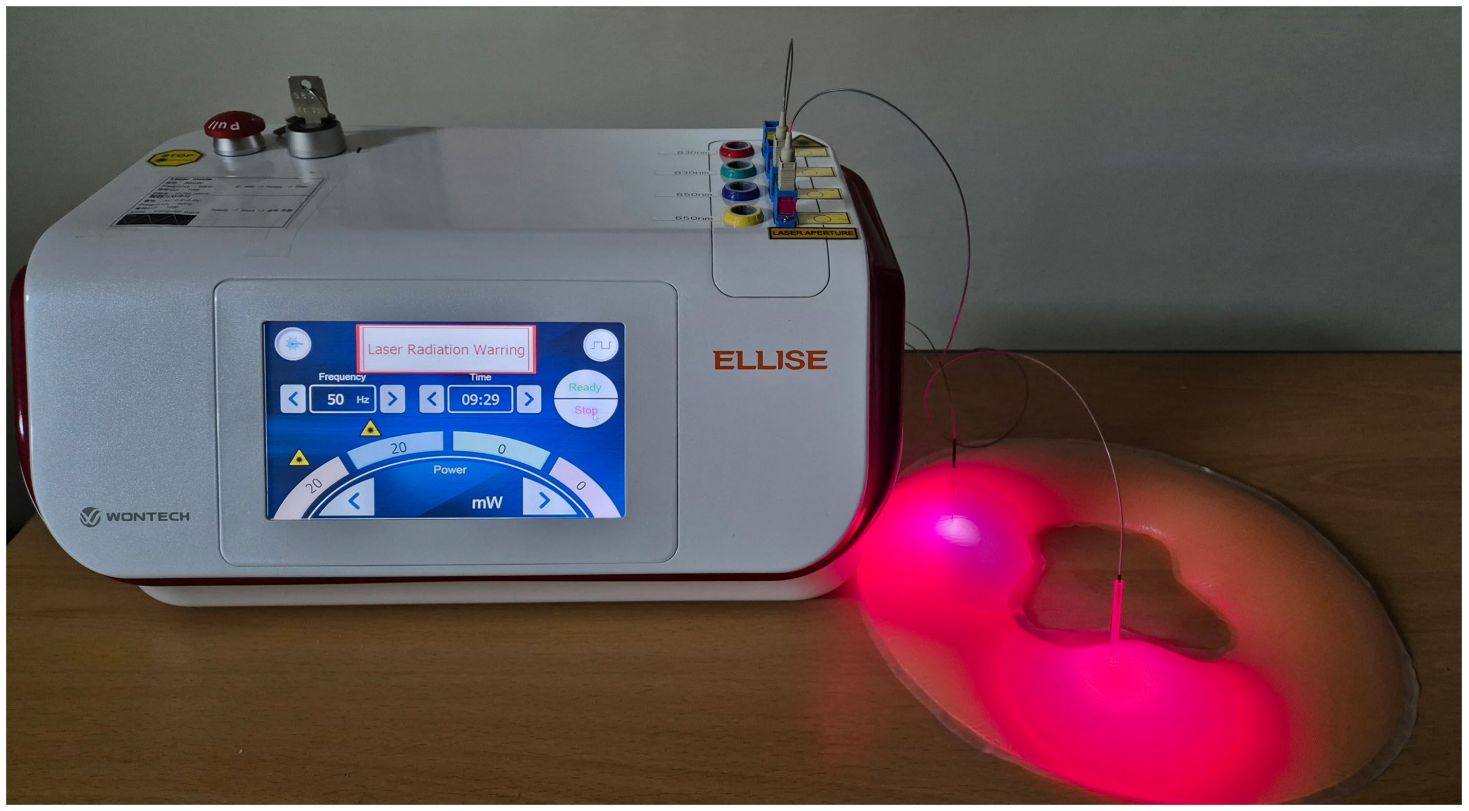
Invasive laser acupuncture device. The device used for invasive laser acupuncture (650 nm wavelength, 50 Hz, 20 mW). The laser fiber is inserted through an acupuncture needle to deliver low-level laser energy directly into the target tissue beneath the skin.

Participants in the experimental group will receive ILA using the Ellise Medical Laser Device (Wontech Co., Ltd., Daejeon, Republic of Korea) (Figure 2), whereas those in the control group will be treated with EA using an ES-160 device (Shinwoo Mediland Co., Ltd., Korea).

The Ellise device delivers 650 nm wavelength laser stimulation through a sterile acupuncture needle integrated with optical fibers. The system consists of a laser output unit, an optical fiber-coupled InGaAIP laser diode, and a disposable needle with embedded optical fibers for precise energy transmission. The stimulation parameters are set to a frequency of 50 Hz, output power of 20 mW, power density of 63.69 W/cm^2^, energy dose of 12 J/point, and energy density of 38,216.56 J/cm^2^ delivered in a pulse-type wave mode.

EA in the control group will be delivered using biphasic alternating current stimulation with an output range of 0.0–5.0 V and a frequency of 50 Hz, based on previous clinical studies on chronic LBP (21). The intensity will be adjusted individually to a level that is perceptible but comfortable for each participant.

Throughout the intervention period (Visit 1–Visit 8), all the participants will be instructed on exercise and self-management techniques to support symptom control. As rescue medication, acetaminophen (500 mg) will be made available to participants experiencing significant pain.

### 2.8 Outcomes

#### 2.8.1 Primary outcomes

The primary outcome measure is the difference in VAS score changes between the experimental and control groups 1 week (±3 days) after the completion of the 4-week treatment period (twice per week, totaling eight sessions) compared to baseline.

#### 2.8.2 Secondary outcomes

Secondary outcomes include the proportion of responders at Visit 9. A responder is defined as a participant who shows a ≥ 30% reduction in VAS score compared to baseline without an increase in the type or dosage of pain medication. This definition follows standard criteria for assessing clinically meaningful improvement in chronic LBP (22, 23). Additional secondary outcomes include changes in VAS scores at the fifth treatment session (Visit 5) and at 8 weeks (±3 days) after treatment completion (Visit 10). Functional disability will be assessed using the ODI, and health-related quality of life will be measured using the EQ-5D-5L. Changes in both scores will be analyzed at Visits 5, 9, and 10 relative to baseline (Visit 1).

### 2.9 Sample size

In a previous study (24) on NSCLBP, the mean change in VAS (± standard deviation) was 9.0 ± 13.10 in the control group and 24.09 ± 14.71 in the treatment group, with a mean difference of 15.09 and a pooled standard deviation of 14.30.

Based on these findings, this study assumes an effect difference of 10 and a standard deviation of 15 for the sample size calculation. A two-sided significance level of 5% and statistical power of 80% were applied with a 1:1 allocation ratio, resulting in a minimum of 36 participants per group. Considering a 20% dropout rate, the final target enrollment is 90 participants, with 45 in each group.


$$\{\frac{2{\left(z_{1-\frac{\alpha }{2}}+z_{\beta }\right)}^{2*}\sigma^{2}}{|\mu_{T}-\mu_{c}|}\}=\{\frac{2{\left(1.96+0.84\right)}^{2*}15^{2}}{10^{2}}\}=35.320\approx 36$$

### 2.10 Statistical analyses

Statistical analysis will be performed by an independent statistician, and group allocation information will be kept blinded until the study is completed. All analyses will be performed using SAS^®^ Version 9.4 with a two-sided test (*α* = 0.05).

The analysis sets are divided into Full Analysis (FA), Per Protocol (PP), and Safety Analysis (SA) sets. The FA set includes the participants who received at least one treatment and underwent an efficacy evaluation. The PP set consists of participants in the FA set who adhered to the study protocol, whereas the SA set includes all participants who received at least one treatment. In this study, the FA set is used as the primary analysis set, whereas the PP set is used for supplementary analysis. The Last Observation Carried Forward (LOCF) method will be used to manage missing values, while the safety assessment will be analyzed without imputation.

Descriptive statistics will be used to summarize demographic and baseline clinical characteristics. Between-group comparisons will be conducted using an independent t-test or Mann–Whitney U test for continuous variables and a chi-square test or Fisher’s exact test for categorical variables.

The efficacy analyses will focus on both primary and secondary outcomes, including changes in VAS, ODI, and EQ-5D-5L scores from baseline to each evaluation time point, analyzed using ANCOVA with baseline values as covariates. When appropriate, repeated-measures ANOVA or paired t-tests (or Wilcoxon signed-rank tests for non-normal data) will be used to assess within-group and between-group changes over time. Safety evaluations will compare the incidence rates of adverse events (AEs) and serious adverse events (SAEs) using the chi-square test or Fisher’s exact test. The frequency, severity, and causal relationship of AEs will be summarized descriptively.

A final analysis will be conducted after study completion without an interim analysis.

### 2.11 Data management and confidentiality

Data management for this study will be conducted by the Korea Institute of Oriental Medicine, and all study-related documents will be assigned unique identification codes to ensure participant anonymity. Personally identifiable information, such as names, will be excluded, and access to identification records will be strictly restricted and permitted only with prior approval from the Institutional Review Board (IRB). An independent data manager will record the data, and clinical research coordinators (CRCs) will enter the information into electronic case report forms (eCRFs), which will be verified by an investigator not involved in the clinical procedures. The data will be securely stored in eCRFs using the myTrial data management system (provided by National Institute of Korean Medicine Development, Republic of Korea), with access protected by a username and password. The data manager and an independent statistician, who have no conflicts of interest with the study, will have full access to the complete database, whereas each institution will be limited to viewing its own data. The central data management center at the Korea Institute of Oriental Medicine will operate independently of the study sponsor and maintain neutrality. Unauthorized access to data will be strictly prohibited unless approved by the IRB. All source data will be securely archived for 3 years after study completion, and written informed consent will be obtained from all participants for the use and sharing of their anonymized data.

### 2.12 Safety assessment and adverse event reporting

All AEs and SAEs will be closely monitored and recorded during the study. Each event will include details such as severity, onset time, duration, potential association with the intervention, management by the CRC, and clinical outcomes. All AEs and SAEs will be reviewed by the investigators and reported to the IRB in accordance with ethical and regulatory requirements. Participants experiencing intervention-related AEs or SAEs will receive appropriate care and compensation according to institutional and legal standards. This safety monitoring process will ensure transparent documentation and reliable assessment of the intervention’s safety profile.

### 2.13 Ethical considerations

This study complies with the Declaration of Helsinki and the Korean Good Clinical Practice (GCP) guidelines and received IRB approval from Dongshin University Gwangju Korean Medicine Hospital (approval no. DSGOH-2024-002, December 16, 2024) and Kyung Hee University Korean Medicine Hospital (approval no. KOMCIRB 2024–12–005-002, April 14, 2025). This study (version 1.0) has been approved by the Ministry of Food and Drug Safety (MFDS) (Medical Device Approval # 1800; November 25, 2024).

## 3 Discussion

NSCLBP is a common musculoskeletal disorder that imposes a socioeconomic burden and impairs an individual’s quality of life, owing to its high prevalence and recurrence rate. This study aims to evaluate the effectiveness of a 650 nm ILA using an invasive laser device (Ellise) in improving pain and function compared with EA, which is widely used in Korean clinical practice.

The Ellise device is expected to exert multifaceted effects, such as pain relief, enhanced tissue healing, improved blood circulation, and anti-inflammatory action. These effects are achieved by combining the biological properties of laser therapy with the mechanical stimulation provided by acupuncture. This suggests the potential for a faster and stronger initial response in patients with NSCLBP. In addition, it may serve as an alternative to EA or conventional acupuncture in Korean medicine and contribute to the expansion of treatment protocols in Korean medicine hospitals and pain clinics.

This clinical trial focuses on short-term improvement in pain; therefore, analyses of long-term outcomes may be limited. In addition, because ILA and EA have different stimulation methods and sensations, complete double-blinding is difficult, and there is a possibility of unblinding between the practitioner and patient. However, this study employed a single-blind design involving an outcome evaluator and data analyst to minimize potential bias. Based on the results of this study, future studies should include a long-term follow-up.

In addition, its applicability to other musculoskeletal pain conditions, such as shoulder and knee pain, should be further explored. Future studies should also evaluate the long-term outcomes of ILA to determine the durability of its therapeutic effects. Ultimately, the development and standardization of a unified treatment protocol across various pain conditions, supported by large-scale confirmatory trials, is anticipated to further validate its safety and efficacy.
